# Germline *MLH1, MSH2* and *MSH6* variants in Brazilian patients with colorectal cancer and clinical features suggestive of Lynch Syndrome

**DOI:** 10.1002/cam4.1316

**Published:** 2018-03-25

**Authors:** Nayê Balzan Schneider, Tatiane Pastor, André Escremim de Paula, Maria Isabel Achatz, Ândrea Ribeiro dos Santos, Fernanda Sales Luiz Vianna, Clévia Rosset, Manuela Pinheiro, Patricia Ashton‐Prolla, Miguel Ângelo Martins Moreira, Edenir Inêz Palmero, Brazilian Lynch Syndrome Study Group, Patrícia Santos Silva, Patrícia Koehler‐Santos, Silvia Liliana Cossio, Cristina Netto, Gustavo Stumpf da Silva, Fernando Regla Vargas, Maria Angélica de Lima, Cristovam Scapulatempo‐Neto, Rui Manuel Reis, André Lopes Carvalho, Carla Pinto, Manuel Rui Teixeira, Danilo Vilela Viana, Benedito Mauro Rossi, Junea Caris Oliveira, Henrique Campos Galvão, Paulo Assumpção, Geraldo Ishak, Sérgio Lima Júnior

**Affiliations:** ^1^ Laboratório de Medicina Genômica Centro de Pesquisa Experimental Hospital de Clínicas de Porto Alegre (HCPA) and Programa de Pós Graduação em Genética e Biologia Molecular Universidade Federal do Rio Grande do Sul (UFRGS) Porto Alegre Brazil; ^2^ Genetics Program Instituto Nacional de Câncer Rio de Janeiro Brazil; ^3^ Molecular Oncology Research Center Barretos Cancer Hospital Barretos Brazil; ^4^ AC Camargo Cancer Center São Paulo Brazil; ^5^ Clinical Genetics Branch Division of Cancer Epidemiology and Genetics Department of Health and Human Services National Cancer Institute National Institutes of Health Bethesda Maryland; ^6^ Núcleo de Pesquisas Oncológicas and Laboratório de Genética Humana e Médica Universidade Federal do Pará Universidade Federal do Pará (UFPA) Belém Brazil; ^7^ Laboratório de Pesquisa em Bioética e Ética na Ciência‐ LAPEBEC ‐ Centro de Pesquisa Experimental Hospital de Clínicas de Porto Alegre Porto Alegre Brazil; ^8^ Serviço de Genética Instituto Português de Oncologia do Porto (IPO Porto) Porto Portugal; ^9^ Barretos School of Health Sciences Dr. Paulo Prata – FACISB Barretos Brazil

**Keywords:** Colorectal cancer, Lynch syndrome, MMR genes

## Abstract

Lynch syndrome (LS) is the most common hereditary colorectal cancer syndrome, caused by germline mutations in one of the major genes involved in mismatch repair (MMR): *MLH1*,*MSH2*,*MSH6* and more rarely, *PMS2*. Recently, germline deletions in *EPCAM* have been also associated to the syndrome. Most of the pathogenic MMR mutations found in LS families occur in *MLH1* or *MSH2*. Gene variants include missense*,* nonsense, frameshift mutations, large genomic rearrangements and splice‐site variants and most of the studies reporting the molecular characterization of LS families have been conducted outside South America. In this study, we analyzed 60 unrelated probands diagnosed with colorectal cancer and LS criteria. Testing for germline mutations and/or rearrangements in the most commonly affected MMR genes (*MLH1, MSH2, EPCAM* and *MSH6*) was done by Sanger sequencing and MLPA. Pathogenic or likely pathogenic variants were identified in *MLH1* or *MSH2* in 21 probands (35.0%). Of these, approximately one‐third were gene rearrangements. In addition, nine variants of uncertain significance (VUS) were identified in 10 (16.6%) of the sixty probands analyzed. Other four novel variants were identified, only in *MLH1*. Our results suggest that *MSH6* pathogenic variants are not common among Brazilian LS probands diagnosed with CRC and that MMR gene rearrangements account for a significant proportion of the germline variants in this population underscoring the need to include rearrangement analysis in the molecular testing of Brazilian individuals with suspected Lynch syndrome.

## Introduction

Lynch Syndrome (LS) is an autosomal dominant cancer predisposition syndrome caused by germline mutations in one of the mismatch repair (MMR) genes and is the cause of approximately 5% of all colorectal cancer (CRC) diagnoses 1. The most frequently affected genes are MutL Homolog 1 (*MLH1*), MutS Homolog 2 (*MSH2*), followed by MutS Homolog 6 (*MSH6*) and Post‐Meiotic Segregation Increased 2 (*PMS2*) 2, 3. Germline mutations in *MLH1* and *MSH2* account for more than 90% of mutations identified in LS families 4. Recently, germline deletions of the last few exons of the Epithelial Cell Adhesion Molecule (*EPCAM*) gene adjacent to *MSH2*, have been also associated to the syndrome. Germline deletions occur in about 1–3% of the Lynch syndrome families and lead to epigenetic silencing of *MSH2*. Germline mutations in *MSH6* and *PMS2* are less common, accounting for about 7–10% and fewer than 5% of all MMR gene mutations, respectively 5, 6.

The classic LS phenotype is characterized by early onset colorectal cancer and an increased risk of extracolonic malignancies, including endometrial, gastric, small bowel, urological tract, ovarian, pancreatic and brain cancers 7. Major differences in lifetime risks of cancer have been reported among MMR mutation carriers, with the highest risk attributed to the presence of a mutation in either *MLH1* or *MSH2*. Cancers in families with an *MSH6* pathogenic variant are usually diagnosed later and cancer risks are lower, with the exception of endometrial cancer 8, 9, 10, 11. Presence of a germline *PMS2* pathogenic variant is associated with the lowest lifetime risk (25–32%) for any Lynch syndrome‐related cancer 12.

Germline variants in the MMR genes of LS patients usually result in loss of nuclear expression of the corresponding gene, which can be identified by immunohistochemistry (IHC). In addition, MMR deficiency results in the accumulation of DNA replication errors, which can be detected as microsatellite instability (MSI), a hallmark of LS 13. The primary indication of MMR germline mutation testing is the presence of a personal and family history of cancer including tumors of the LS spectrum 14. Using this approach, the Amsterdam criteria allow the clinical diagnosis of LS and although specific, show little sensitivity 15. The Bethesda criteria, on the other hand, identify individuals with CRC or other LS tumors who should have their cancers tested for evidence of MMR deficiency using IHC and/or MSI 16, and if deficiency is identified, MMR germline mutation testing is warranted. Prior probability of carrying an MMR germline mutation can be estimated using mathematical prediction models such as the PREMM1,2,6 model 17, 18; these models are useful in the indication of genetic testing.

Most of the pathogenic MMR mutations found in LS families are missense, nonsense and frameshift mutations, whereas large genomic rearrangements and splice‐site variants constitute <10% of the alterations 8, 19, 20, 21, 22, 23. Furthermore, germline epimutations and promoter variations were reported in some LS families 24, 25 and founder mutations have been identified in some populations, including the Finns, Newfoundlanders, Portuguese and Ashkenazi Jews, in which they are responsible for a significant proportion of Lynch syndrome cases 26.

Most of the studies reporting the molecular characterization of LS families have been conducted in North America, Europe and Asia 27. Only a few studies describe the prevalence and type of MMR mutations in Latin America and more specifically in Brazil 28, 29, 30, 31, 32, 33, 34, 35. Therefore, the aim of this study is to describe the frequency and profile of germline variations in the most commonly affected MMR genes, *MLH1*,* MSH2* and *MSH6* genes in a group of Brazilian patients with colorectal cancer fulfilling the Amsterdam or Bethesda criteria for LS and correlate the presence of mutations with clinical aspects and mutation prediction.

## Methods

### Patients

Five medical centers who provide cancer genetics services in three regions of Brazil participated in the study. Unrelated, cancer‐affected probands from hereditary cancer registries of the participating institutions, who fulfilled either Amsterdam or Bethesda criteria were included after providing informed consent. Genomic DNA was isolated from peripheral blood lymphocytes using standard protocols. Sixty patients were recruited from July 2011 to July 2013 at Hospital de Clínicas de Porto Alegre (*n* = 18) in the Southern region of Brazil, Hospital de Câncer de Barretos (*n* = 13), Hospital AC Camargo (*n* = 14) and Instituto Nacional de Câncer (*n* = 8) in the Southeastern region, and from Hospital João de Barros Barreto (*n* = 7) in the Northern (Amazonian) Region of Brazil. Pedigrees, pathology reports and additional relevant clinical information were obtained from review of medical records and patient interviews (Table S1). The study was approved in all participating institutions by their institutional ethics committees (CAAE – 0254.1.001.007‐11). Patients included in this study were also included, with additional LS probands, in a separate methodologic study aiming at the validation of a next‐generation sequencing (NGS) protocol 36.

### Prior probability of carrying a mutation using the PREMM1,2,6 score

Detailed clinical information necessary for generating the PREMM1,2,6 score was extracted from pedigrees for each proband. The following data were used to derive a unique PREMM1,2,6 score for each study participant using a web‐based tool (http://premm.dfci.harvard.edu/): (1) proband‐specific variables, including gender, occurrence and age of CRC, endometrial and/or other Lynch syndrome‐associated cancer diagnoses; extracolonic cancers, including those of the ovary, stomach, kidney, ureter, bile duct, small bowel, brain (glioblastoma multiforme), pancreas, or sebaceous glands; (2) family history related variables, including number of relatives with CRC, endometrial cancer, or other Lynch syndrome‐associated cancers; relationship to proband (first‐ vs. second‐degree); minimum age at diagnosis of each cancer in the family.

### *MLH1, MSH2* and *MSH6* genotyping

Sanger sequencing was performed on ABI PRISM 3130XL or ABI3500 Genetic Analyzer (Applied Biosystems, Foster City, CA). All mutation‐positive samples were confirmed in a second independent analysis. Primers used for PCR amplification and *MLH1* (NM_000249.3) sequencing were previously described by Hegde et al. For *MSH2* (NM_000251.2), primers used were previously described by Becket al. 38 and Zahary et al. 39, and for *MSH6* (NM_000179.2), the primers used were previously described by Hegde et al. 37.

### Multiplex ligation probe amplification (MLPA)

To detect large genomic rearrangements in *MLH1*,* MSH2*, and *MSH6*, the SALSA MLPA Kits P003‐C1 and P072‐C1 were employed according to the manufacturer's instructions (MRC Amsterdam Holland, the Netherlands). Amplification products were identified using an ABI3500 genetic analyser (Applied Biosystems) and results interpreted using the software Coffalyser.net. Commercial DNA sample was used as reference (Promega, Madison, WI).

### Chromosome Microarray

To confirm large rearrangements found by MLPA, we used the chromosomal microarray technique CytoScan HD (Affymetrix), according to the manufacturer's protocol. The high‐density, whole‐genome CytoScan Array includes 2.69 million markers for copy number analysis, which are representative of DNA sequences distributed throughout the genome. Chromosome Analysis Suite software (ChAS software 3.1) was used to analyze and visualize microarray data. Deletions and duplications with more than 50 markers involved and size of 250 bp or more were considered in ChAS analysis. The following parameters were also considered: mean marker distance, copy number state, smooth signal and log2 ratio. Data were compared with the Database of Genomic Variants (DGV‐http://dgv.tcag.ca/dgv/app/home), affymetrix DGV (a database created by affymetrix that includes deletions and duplications found by Cytoscan HD) and common artifacts database (a database created by affymetrix that includes artifacts commonly found by Cytoscan HD, used to exclude this type of alteration).

### Variant nomenclature and classification

Mutation nomenclature followed the Human Genome Variation Society (HGVS) guidelines (http://www.hgvs.org/mutnomen/recs.html). Variants were classified according LOVD (http://www.lovd.nl/3.0/home) and NCBIClinVar (https://www.ncbi.nlm.nih.gov/clinvar/). In addition, for variants of unknown significance (VUS) we also assessed the ACMG classification system 40. Non‐described variants were classified by ACMG and in silico pathogenicity predictors: SIFT (http://www.sift.jcvi.org), Poly‐Phen‐2 (http://www.genetics.bwh.harvard.edu/pph2) and AlignGVGD (http://www.agvgd.hci.utah.edu), which assess the potential effects of missense variants on protein function. SPSS v.18 was used for data handling and descriptive analysis. Population frequency variants were searched in 1000 Genomes browser (http://www.ncbi.nlm.nih.gov/variation/tools/1000genomes/) and if data was missing in EXAC (http://exac.broadinstitute.org/) was consulted.

### Mutation prevalence and PREMM1,2,6 performance

Estimated and observed mutation prevalence among probands with either Amsterdam or Bethesda criteria were compared using Binomial Test. To evaluate the sensibility and specificity of the PREMM1,2,6 model in our sample, receiver operating characteristic (ROC) curves were constructed by choosing cutpoints and computing the sensitivity against specificity. Furthermore, we used Youden Index (J), another main summary statistic of the ROC curve, which defines the maximal potential effectiveness of accuracy of PREMM.

## Results

Of the 60 patients recruited, 51.7% were female, all had been diagnosed with colorectal cancer (CRC) and the median age at first cancer diagnosis was 40.8 years (range 19–81 years) (Table 1). Estimated prior probability of mutation using the PREMM1,2,6 model was 53.4% and 12.2% in patients fulfilling the Amsterdam and Bethesda criteria, respectively. Sequencing of the coding regions of *MLH1*,* MSH2* and *MSH6* was successful in all patients and MLPA was conclusive in 58 and 59 patients for *MLH1/MSH2* and *MSH6* rearrangement analysis, respectively.

**Table 1:** Clinical features of the 60 probands included in the study

	*N* (%)	Median age at cancer diagnosis years (range)	Overall PREMM1,2,6 score (Avg %)	PREMM1,2,6 score per gene (Avg %)
Sex				
Female	31 (51.7)	–		
First cancer diagnosis		40.8 (19–81)		
Clinical criteria				
Amsterdam	27 (45)	43 (21–70)	53.4	*MLH1* 24.1
				*MSH2* 23.9
				*MSH6* 1.7
Bethesda	33 (55)	39 (20–81)	12.2	*MLH1* 5.7
				*MSH2* 5.2
				*MSH6* 1.4

**Figure 1:**
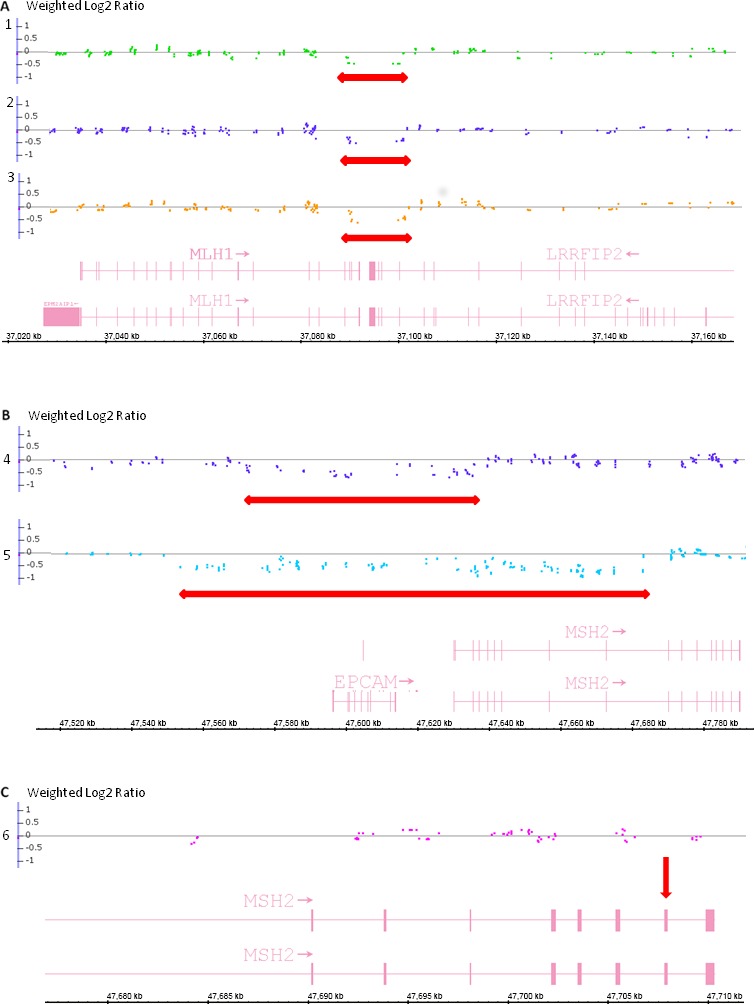
Chromosome microarray results of the MLPA positive patients. Each patient is represented by a gray line (numbers 1, 2, 3, 4, 5 and 6). The dots represent the Weighted Log2 Ratio measured to each marker in chromosome microarray. Dots on the gray line represent markers with a Weighted Log2 Ratio = 0, indicating that no copy number variation occurs; markers with a reduced signal intensity are represented by dots under the line; Weighted Log2 Ratio = −0.5 represents a heterozygous deletion; Weighted Log2 Ratio = −1 represents a homozygous deletion; positive values for Weighted Log2 Ratio represent duplications. Arrows represent the extension of the corresponding deletions. (A) Three patients showed *MLH1* heterozygous deletion of exons 17, 18 and 19. In chromosome microarray, we observed that this deletion extended to the neighbor gene *LRRFIP2,* exons 16–19. The deletion has the same extension in the three patients, encompassing 12,050 base pairs (the first deleted marker is at position chr3g.37090000 and the last is at position chr3g.37102050). (B) Two patients showed heterozygous *EPCAM/MSH2* deletion in MLPA analysis. In chromosome microarray, the extension of the heterozygous deletion is different between the two patients. The first patient (number 4) has *EPCAM* complete deletion and *MSH2* deletion of exons 1–4, encompassing 58,000 base pairs (the first deleted marker is at position chr2g.47582000 and the last is at position chr2g.47640000). The second patient (number 5) has *EPCAM* complete deletion and *MSH2* deletion of exons 1–8, encompassing 133,920 base pairs (the first deleted marker is at position chr2g.47547920 and the last is at position chr2g.47682000). (C) One patient harbored a deletion of *MSH2* exon 16 in MLPA analysis. Chromosome microarray is designed to detected larger deletions, since many exons may not be covered by a marker; a zoom in *MSH2* exon 16 is indicated by the arrow, and no marker is present to detect copy number variation for this exon, as observed in the gray line above it. For this reason, this deletion was not confirmed by microarray technique, but we confirmed that no point mutations are present in this exon, which could result in MLPA probe hybridization failure. No other significant alterations were found in other chromosome regions in the six analyzed patients.

Overall, pathogenic variants were identified only in the *MLH1* and *MSH2* genes including two *MSH2/EPCAM* deletions. Of the 60 probands tested, 21 (35%) harbored a pathogenic or likely pathogenic mutation and 15 of these mutations (71.4%) were identified by gene sequencing (Table 2). Gene rearrangements were present in 6 (10.3%) of the 58 probands with conclusive MLPA results and included 3 large deletions in *MLH1*, 2 in *EPCAM/MSH2,* and 1 in *MSH2* (Fig. 1). The deletion identified in *MLH1*, encompassing exons 17, 18 and 19 in three patients had been previously described by Pinheiro et al. as a founder mutation in Northern Portugal 41. This deletion was confirmed in the three patients by Cytoscan HD. By this technique, the deletion was found to start at *MLH1* exon 17 and extends to the adjacent gene *LRRFIP2*, spanning 12050pb. The two deletions involving *EPCAM/MSH2* included *MSH2* exons 1–4 in one case and *MSH2* exons 1–8 in the other. The same *EPCAM* exons were absent in both deletions. Both *EPCAM/MSH2* deletions were found by Cytoscan HD with the same extension found by MLPA. One patient harbored a deletion of *MSH2* exon 16, sequencing through this region failed to identify a sequence variant in the region complementary to the exon 16 MLPA probe, which could have resulted in probe hybridization failure and false‐negative deletion results 42. As this single exon deletion is too small to be detected by Chromosomal microarray, it was not confirmed by this technique.

**Table 2:** Pathogenic variants identified in the 60 individuals analyzed

Gene	ID	Nucleotide	Consequence	Exon	Reported as		Probands affected
					CLINVAR	LOVD	
*MLH1*	rs267607778	c.677 + 1G>A	Aberrant Splicing/Splice donor variant	NA	Likely pathogenic	Likely pathogenic	1
*MLH1*	rs63751711	c.677G>A	p.Arg226Gln	8	Pathogenic	Pathogenic	2
*MLH1*	–	c.791‐4_795delTTAGATCGT	Frameshift	10	ND	Pathogenic	1
*MLH1*	rs63750316	c.1276C>T	p.Gln426Ter	12	Pathogenic	Pathogenic	1
*MLH1*	rs587778949	c.1853delAinsTTCTT	p.Lys618IlefsTer4	16	Pathogenic	Pathogenic	1
*MLH1*	rs63751310	c.1975C>T	p.Arg659Ter	17	Pathogenic	Pathogenic	1
*MSH2*	rs63750704	c.388_389del_CA	p.Gln130ValfsTer2	3	Pathogenic	Pathogenic	1
*MSH2*	rs193922376	c.942 + 3A>T	Aberrant Splicing/Exon loss	NA	Pathogenic	Pathogenic	1
*MSH2*	rs587779067	c.1046C>G	p.Pro349Arg	6	Pathogenic	Pathogenic	1
*MSH2*	rs267607996	c.2021G>A	p.Gly674Asp	13	Likely pathogenic	Likely pathogenic	1
*MSH2*	rs63750636	c.2131C>T	p.Arg711Ter	13	Pathogenic	Pathogenic	1
*MSH2*	rs587779139	c.2152C>T	p.Gln718Ter	13	Pathogenic	Pathogenic	3

ND, Not described; NA, Not applicable.

The observed frequency of pathogenic or likely pathogenic mutations was higher in probands fulfilling the Amsterdam criteria (30%) when compared to those fulfilling Bethesda criteria (5%) and all but one of the six probands carrying gene rearrangements fulfilled Amsterdam criteria. Average estimated prior probability of mutation in the Amsterdam group of patients was different than the observed mutation prevalence (53.4% vs. 30%; *P* = 0.022). Although a difference was also observed in the Bethesda group of patients (12.2% vs. 5%) it did not reach statistical significance.

In addition, nine variants of uncertain significance (VUS) were identified in 10 (16.6%) of the sixty probands analyzed by sequencing (Table 3) and four novel variants were identified in *MLH1*. These novel variants were frameshift alterations, and were classified by ACMG in likely pathogenic (p.Tyr561LeufsTer7, p.Ile75MetfsTer17), pathogenic (p.Leu296PhefsTer10) and uncertain significance (p.Met621IlefsTer16).

**Table 3:** Variants of uncertain significance (VUS) identified in the 60 individuals analyzed

Gene	ID	Nucleotide	Consequence	Exon	Reported as			Proband affected	Population frequency
					CLINVAR	LOVD	ACMG		
*MLH1*	rs41295282	c.277A>G	p.Ser93Gly	3	VUS	VUS	VUS	1	G = 0.000033^a^
*MLH1*	rs587781750	c.1007G>A	p.Gly336Asp	11	VUS	ND	VUS	1	ND
*MLH1*	rs35001569	c.1852A>G	p.Lys618Glu	16	VUS	VUS	LB	1	G = 0.0032^b^
*MLH1*	rs63750242	c.2027T>C	p.Leu676Pro	18	VUS	VUS	LB	1	ND
*MSH2*	rs1057524909	c.2078G>A	p.Cys693Tyr	13	VUS	VUS	LB	1	A = 0.0002^b^
*MSH6*	rs555209664	c.2006T>C	p.Ile669Thr	4	VUS	VUS	VUS	1	C = 0.0002^b^
*MSH6*	rs542848931	c.719G>A	p.Arg240Gln	4	VUS	ND	LB	2	A = 0.0002^b^
*MSH6*	rs41557217	c.633A>C	p.Glu221Asp	4	VUS	VUS	VUS	1	C = 0.0006^a^
*MSH6*	rs63750554	c.3772C>G	p.Gln1258Glu	8	VUS	ND	VUS	1	G = 0.0002^b^

LB, Likely benign; ND, Not described; VUS, Variant of uncertain significance.

a ExAC.

b 1000 genomes.

**Figure 2:**
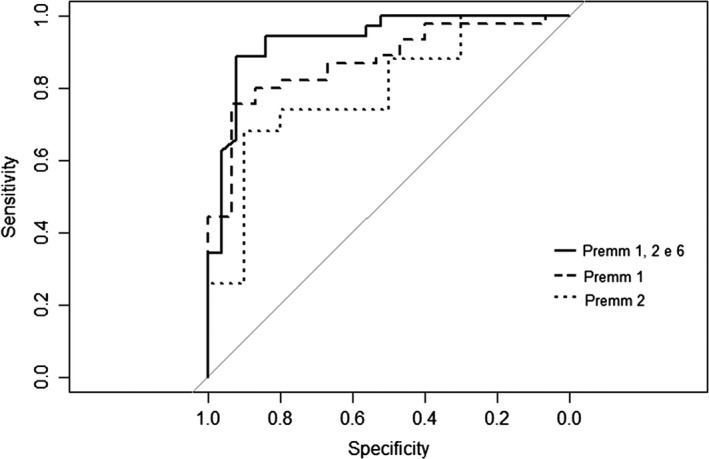
ROC Curve the MMR mutation risk prediction model PREMM1,2,6 in a sample of Brazilian Lynch syndrome probands.

Regarding performance of the PREMM1,2,6 score in this sample, we observed an AUC of 0.93 (95% confidence interval [CI], 0.87–1.00) for PREMM1,2,6, (Fig. 2), 0.87 (95% CI, 0.778–0.967) for PREMM1 and 0.79 (95% CI, 0.643–0.941) for PREMM2 (Table 4). The Youden index was 19.5% of the PREMM, with 0.92 sensitivity and 0.88 of specificity.

**Table 4:** Area under the Receiver‐operator characteristic (ROC) curve of the PREMM1,2,6 model

	AUC	SE	CI 95%	Sensitivity	Specificity
PREMM1	0.87	0.05	0.778–0.967	0.93	0.71
PREMM2	0.79	0.08	0.643–0.941	0.90	0.68
PREMM1,2,6	0.93	0.03	0.87–1.000	0.92	0.89

AUC, area under the ROC curve; SE, standard error; CI 95%, confidence interval 95%.

## Discussion

Even decades after the identification of the most commonly mutated genes in Lynch syndrome, knowledge about the prevalence and spectrum of deleterious mutations in Brazilian families remains incomplete. Germline mutation testing in Brazil has only recently become available for patients with private insurance and remains largely unavailable in the public health care system. Thus, the aim of our study was to perform a comprehensive analysis of the most commonly affected genes in LS (*MLH1*,* MSH2*,* MSH6* and *EPCAM*) and assess performance of a mutation prediction tool in this group of patients.

As expected from previous findings in Brazilian patients and in the literature, which describe *MLH1* and *MSH2* as the mutant genes in up to 90% of the mutation‐positive LS families 1, 2, 3, 4, 31, 32, 33, 34, 35, 36, these two genes harbored all of the deleterious mutations found in our analysis. In addition, in the *MSH6* gene we only identified VUS. Usually, *MSH6* mutations account for about 7–10% of the pathogenic mutations in LS and in our study, the absence of mutations may be due to features of the group of patients under study (colorectal cancer patients with a first cancer diagnosis under the age of 45 years in average) and/or due to our small sample size. In a large genotype‐phenotype correlation study, mutations in *MLH1* and *MSH2* were highly prevalent in patients with a positive family history and cancer diagnosis at young age, whereas the prevalence of mutations in *MSH6* and other genes were increased among isolated cancer cases and families with a higher age of cancer onset 43.

Interestingly, among the 21 probands with deleterious mutations, 28.6% harbored a gene rearrangement, underscoring the need to include rearrangement screening in the routine molecular diagnosis of Lynch syndrome in Brazilian patients. In particular, combined deletions of several exons of *MSH2/EPCAM* occurred in two probands, representing about 3% of all patients analyzed, and is in accordance with the 1–3% expect prevalence of this type of mutation in LS families reported to date 6. In previous mutation characterization studies, rearrangements also represent a significant fraction of variants in LS genes, but usually not as high as the observed in our series, and they most commonly affect *MSH2* [44, 45]. In addition to *MSH2/EPCAM* rearrangements, we identified a large *MLH1* deletion encompassing exons 17–19 in 3 probands. This rearrangement was previously described by Pinheiro et al. 41 in Portuguese individuals and extends to exons 26, 27, 28, and 29 of the adjacent *LRRFIP2* gene. As expected, all 3 probands referred Portuguese ancestry.

Also as expected, we identified most mutations in patients with clinical criteria for LS (Amsterdam criteria) when compared to the group of patients with less striking phenotypes (those with Bethesda criteria). Only 3 of the 21 probands with deleterious mutations did not fulfill Amsterdam criteria, again in agreement with previous reports from other populations 35, 46, 47. However, what is striking is that overall mutation prevalence, even in the Amsterdam group of patients was lower than expected. Several studies have demonstrated that mutation prevalence in this group of patients is in the range of 45–55%. Here, only 35% had a pathogenic or likely pathogenic mutation despite comprehensive analysis of the three most commonly affected genes. A similar mutation prevalence was observed in another study involving Brazilian patients, which detected mutations in 38.8% of the probands tested for *MLH1*,* MSH2*,* MSH6*,* PMS2* and *PMS1* 35. There are a few case reports of germline variants in other MMR or MMR‐related genes such as *MLH3*,* MSH3*,* EXO1*, or *TGFBR2* among LS families. However, the clinical significance, of most of these variants has not yet been determined and the clinical utility of mutation testing of these genes remains unestablished 48, 49.

Variants of uncertain significance (VUS) are currently identified in 20–30% of LS probands undergoing MMR gene testing 50. Using the ACMG variant classification system, we identified VUS only in 05 (8.3%) of the 60 probands, which is below VUS prevalence data in previously published studies. This system allows variant classification that is less subjective and fewer conflicting results on pathogenicity are expected. In addition to the ACMG classification system we also assessed variant classification in two major databases, ClinVar and LOVD. Considering these databases our results are similar to the literature (VUS prevalence of 16.6%) (Table 3). These inconclusive classifications are a challenge when it comes to medical management decisions 50. The presence of the same VUS in other affect relative may contribute to the classification of a VUS. Although segregation analysis within a family of a variant carrier can be very helpful to understand pathogenicity, it is often a challenging approach, since in many families relatives are either deceased or unavailable for testing. Presence of a second, clearly pathogenic variant in the same MMR gene and especially if observed in trans, is another feature that may help in classification of a VUS, since the phenotype of carrying one mutant allele (Lynch syndrome) is very different from the phenotype of bi‐allelic mutation carriers (Constitutional Mismatch Repair Deficiency – CMRD – Syndrome 51, 52. In the absence of a CMRD phenotype, co‐occurrence of a VUS with a pathogenic mutation is suggestive of its non‐pathogenicity. Among the 21 probands with pathogenic variants identified here, four had no CMRD features and had one or more VUS (*MSH6* p.Arg240Gln, p.Gln1258Glu) *MLH1* (p.Lys618Glu), suggesting that the VUS are not pathogenic.

Finally, we identified four novel *MLH1* frameshift variants which were classified by the ACMG variant classification system (Table 5). As part of this classification, in silico prediction tools assessed two of the variant (p.Leu296Phefs and p.Ile75Metfs) as pathogenic variants. Pathogenicity predictions of the other two (p.Tyr561Leufs, p.Met621Ilefs) indicated contradictory results. Such contradictory results are not uncommon, and therefore in silico predictions should not be used alone to classify any novel variant. Although the presence of a frameshift variant within the coding region of a given gene is a strong indicator of pathogenicity, additional evidence is desirable to define its functional impact and may include intra‐familial variant segregation analysis, population frequency and in vivo/in vitro functional studies 40. Although helpful, results of microsatellite instability (MSI) and loss of MMR protein expression detected by IHC in these four cases also did not provide definitive answers regarding pathogenicity (data not shown).

**Table 5:** Novel variants identified in *MLH1* sequencing

Gene	Nucleotide	Consequence	Exon	Reported as			ACMG	Number of families
				Polyphen	SIFT	GVGD		
*MLH1*	c.888_890delAGAinsC	p.Leu296Phefs	11	1.00	0.00	Class C0	Pathogenic	1
*MLH1*	c.1681dupT	p.Tyr561Leufs	15	0.997	0.00	Class C35	Likely pathogenic	1
*MLH1*	c.1863delG	p.Met621Ilefs	16	0.644	0.11	Class C0	Uncertain significance	1
*MLH1*	c.225delT	p.Ile75Metfs	3	1.00	0.00	Class C0	Likely pathogenic	1

Polyphen = 0: benign 1: pathogenic/SIFT < 0.05 pathogenic/GVGD = Class C65: most likely to interfere with function; Class C0 less likely to interfere with function.

Regarding the analysis of accuracy of the PREMM1,2,6 in this sample of Brazilian LS patients, AUC was 0.93, indicating very good discrimination for the model. A recent study performed in Latin America observed that the AUC for the PREMM1,2,6 model was 0.846 and that other similar mutation prediction tools (Barnetson, MMRpro and Wijnen models) presented similar AUC 31. In that study, the sensitivity of PREMM1,2,6 was 0.74, and the specificity was 0.82. Moreover, the authors also performed different risk scenarios of PREMM1,2,6 probabilities, and from their data, with 10% and 20% PREMM1,2,6 thresholds, sensitivity/specificity of the model reached 0.90/0.54, and 0.67/0.85, respectively. In the present study, we used Younden's Index to determine the point of optimal performance of the PREMM1,2,6 model, which has been used in different studies for this end [53, 54]. Based on our data, if we would have to choose a threshold to refer the patient for genetic testing, it would be around 20% PREMM probability. Clearly, this is a limited study with a small sample size and with a distinct population and recruitment criteria than used in other studies. Therefore, it is difficult to compare our results with previous studies as well as extrapolate results for the general population. However, this is the type of study needed to better define which clinical criteria should be used in the establishment of public policies for genetic testing and management of risk in a clinical setting.

Comprehensive analysis of *MLH1*,* MSH2*,* MSH6* and *EPCAM* genes in this series of Brazilian families with suspected Lynch syndrome shows that *MLH1* and *MSH2* are the genes most commonly affected. Furthermore, gene rearrangements were responsible for a significant proportion of the pathogenic mutations identified, thus confirming the need for always including rearrangement detection methods in the molecular diagnostic strategy of Lynch syndrome in this population. Finally, although the PREMM1,2,6 model performed well in this sample of Brazilian patients with LS in depth phenotypic characterization of a larger series of LS mutation carriers and non‐carriers is important to instrument the construction of mutation prediction tools for LS in this population. This study contributes to the genotypic characterization of LS in Brazil, which is relevant for genetic counseling, diagnosis and cancer prevention.

## Conflict of Interest

None declared.

## Supporting information


**Table S1.** Clinical features of the 60 unrelated probands included in the study.

## Appendix 1

### Brazilian Lynch Study Group

Patrícia Santos Silva, Patrícia Koehler‐Santos, Silvia Liliana Cossio, Cristina Netto, Gustavo Stumpf da Silva (Laboratório de Medicina Genômica, Centro de Pesquisa Experimental, Hospital de Clínicas de Porto Alegre (HCPA) and Programa de Pós Graduação em Genética e Biologia Molecular, Universidade Federal do Rio Grande do Sul (UFRGS), Porto Alegre, Brazil); Fernando Regla Vargas, Maria Angélica de Lima (Genetics Program Instituto Nacional de Câncer, Rio de Janeiro, Brazil); Cristovam Scapulatempo‐Neto, Rui Manuel Reis, André Lopes Carvalho (Molecular Oncology Research Center, Barretos Cancer Hospital, Barretos, Brazil); Carla Pinto, Manuel Rui Teixeira (Serviço de Genética, Instituto Português de Oncologia do Porto (IPO Porto), Porto, Portugal); Danilo Vilela Viana, Benedito Mauro Rossi Junea Caris Oliveira, Henrique Campos Galvão (Oncogenetics Department, Barretos Cancer Hospital, Barretos, Brazil). Paulo Assumpção, Geraldo Ishak, Sérgio Lima Júnior (Núcleo de Pesquisas Oncológicas, Universidade Federal do Pará e Serviço de Cirurgia Geral e do Aparelho Digestivo, Hospital Universitário João Barros Barreto, Universidade Federal do Pará).
