# Supplementary material for: Germline MLH1, MSH2 and MSH6 variants in Brazilian patients with colorectal cancer and clinical features suggestive of Lynch Syndrome
Source: Cancer Med. 2018 Mar 25;7(5):2078–88. doi: 10.1002/cam4.1316 (PMC5943474; doi:10.1002/cam4.1316)
Supplement: Supplementary file 1 — Table S1. Clinical features of the 60 unrelated probands included in the study. [file CAM4-7-2078-s001.docx]

**Supplementary table 1:** Clinical features of the 60 unrelated probands included in the study.

| **Institution** | **Identification** | **Criteria** | **Sex** | **Age at first cancer diagnosis** | **Tumor site** | **Number of first degree relatives with LS tumors** | **Number of generations with LS tumors** |
| --- | --- | --- | --- | --- | --- | --- | --- |
| 1 | 1 | Amsterdam II | F | 51 | Ascending colon | 2 | 3 |
| 1 | 2 | Amsterdam II | M | 52 | Ascending colon | 2 | 4 |
| 1 | 3 | Amsterdam I | M | 48 | Transverse colon | 2 | 3 |
| 1 | 4 | Amsterdam II | F | 42 | Transverse colon | 2 | 3 |
| 1 | 5 | Amsterdam I | M | 37 | Ascending colon | 2 | 2 |
| 1 | 6 | Amsterdam II | M | 29 | Rectosigmoid colon | 1 | 3 |
| 1 | 7 | Bethesda | M | 52 | Rectosigmoid colon | 1 | 3 |
| 1 | 8 | Bethesda | F | 44 | Ascending colon | 0 | 2 |
| 1 | 9 | Bethesda | F | 42 | Ascending colon | 1 | 2 |
| 1 | 10 | Bethesda | F | 48 | Rectosigmoid colon | 0 | 1 |
| 1 | 11 | Bethesda | M | 31 | Rectosigmoid colon | 1 | 3 |
| 1 | 12 | Bethesda | F | 35 | Rectosigmoid colon | 0 | 1 |
| 1 | 13 | Amsterdam I | M | 38 | Transverse colon | 3 | 2 |
| 1 | 14 | Amsterdam II | F | 30 | Transverse colon | 0 | 2 |
| 1 | 15 | Amsterdam I | F | 46 | Ascending colon | 5 | 3 |
| 1 | 16 | Amsterdam II | F | 34 | Rectosigmoid colon | 1 | 3 |
| 1 | 17 | Amsterdam II | F | 36 | Rectosigmoid colon | 1 | 3 |
| 1 | 18 | Amsterdam II | F | 50 | Rectosigmoid colon | 5 | 3 |
| 2 | 19 | Bethesda | F | 28 | Rectosigmoid colon | 0 | 2 |
| 2 | 20 | Amsterdam II | F | 42 | Ascending colon | 2 | 2 |
| 2 | 21 | Bethesda | M | 39 | Transverse colon | 0 | 2 |
| 2 | 22 | Bethesda | F | 46 | Ascending colon | 1 | 2 |
| 2 | 23 | Amsterdam I | M | 39 | Ascending colon | 3 | 3 |
| 2 | 24 | Bethesda | M | 19 | Rectosigmoid colon | 0 | 1 |
| 2 | 25 | Amsterdam II | M | 70 | Ascending colon | 3 | 3 |
| 2 | 26 | Bethesda | M | 32 | Transverse colon | 1 | 3 |

**Supplementary table 1: Continuation**

| **Institution** | **Identification** | **Criteria** | **Sex** | **Age at first cancer diagnosis** | **Tumor site** | **Number of first degree relatives with LS tumors** | **Number of generations with LS tumors** |
| --- | --- | --- | --- | --- | --- | --- | --- |
| 3 | 27 | Bethesda | F | 24 | Rectosigmoid colon | 0 | 2 |
| 3 | 28 | Bethesda | F | 60 | Colorectal,Unspecified site | 2 | 2 |
| 3 | 29 | Bethesda | F | 56 | Ascending colon | 0 | 1 |
| 3 | 30 | Bethesda | M | 46 | Ascending colon | 0 | 1 |
| 3 | 31 | Bethesda | F | 29 | Ascending colon | 0 | 1 |
| 3 | 32 | Amsterdam II | M | 45 | Ascending colon | 1 | 2 |
| 3 | 33 | Bethesda | M | 44 | Rectosigmoid colon | 0 | 1 |
| 3 | 34 | Amsterdam II | F | 34 | Ascending colon | 1 | 3 |
| 3 | 35 | Bethesda | M | 35 | Colorectal,Unspecified site | 1 | 2 |
| 3 | 36 | Amsterdam II | M | 61 | Colorectal,Unspecified site | 2 | 3 |
| 3 | 37 | Amsterdam II | F | 53 | Ascending colon | 1 | 2 |
| 3 | 38 | Amsterdam I | M | 39 | Colorectal,Unspecifieds ite | 8 | 2 |
| 3 | 39 | Bethesda | F | 81 | Ascending colon | 5 | 2 |
| 3 | 40 | Amsterdam II | M | 21 | Ascending colon | 1 | 3 |
| 4 | 41 | Amsterdam I | F | 46 | Ascending colon | 4 | 3 |
| 4 | 42 | Bethesda | F | 35 | Rectosigmoid colon | 0 | 1 |
| 4 | 43 | Bethesda | M | 34 | Rectosigmoid colon | 0 | 1 |
| 4 | 44 | Bethesda | F | 28 | Ascending colon | 0 | 1 |
| 4 | 45 | Bethesda | F | 30 | Colorectal,Unspecified site | 0 | 1 |
| 4 | 46 | Bethesda | F | 52 | Colorectal,Unspecified site | 1 | 2 |
| 4 | 47 | Bethesda | F | 38 | Rectosigmoid colon | 0 | 1 |
| 5 | 48 | Bethesda | M | 51 | Rectosigmoid colon | 1 | 1 |
| 5 | 49 | Bethesda | F | 34 | Transverse colon | 0 | 3 |
| 5 | 50 | Amsterdam II | M | 49 | Colorectal,Unspecifieds ite | 1 | 3 |
| 5 | 51 | Bethesda | F | 28 | Rectosigmoid colon | 0 | 3 |
| 5 | 52 | Bethesda | F | 22 | Rectosigmoid colon | 0 | 1 |
| 5 | 53 | Bethesda | M | 36 | Rectosigmoid colon | 0 | 1 |
| 5 | 54 | Bethesda | M | 20 | Rectosigmoid colon | 1 | 2 |
| 5 | 55 | Bethesda | M | 45 | Rectosigmoid colon | 0 | 2 |

**Supplementary table 1: Continuation**

| **Institution** | **Identification** | **Criteria** | **Sex** | **Age at first cancer diagnosis** | **Tumor site** | **Number of first degree relatives with LS tumors** | **Number of generations with LS tumors** |
| --- | --- | --- | --- | --- | --- | --- | --- |
| 5 | 56 | Amsterdam I | M | 43 | Ascending colon | 2 | 3 |
| 5 | 57 | Bethesda | F | 44 | Ascending colon | 0 | 3 |
| 5 | 58 | Amsterdam II | M | 50 | Colorectal,Unspecified site | 4 | 2 |
| 5 | 59 | Amsterdam I | M | 53 | Descending colon | 3 | 2 |
| 5 | 60 | Amsterdam II | M | 23 | Ascending colon | 0 | 4 |

Hospital de Clínicas de Porto Alegre: 1; Instituto Nacional de Câncer: 2; Hospital AC Camargo: 3; Hospital João de Barros Barreto: 4; Hospital de Câncer de Barretos: 5.
